# A role for RNA stability in the induction of HTLV-1 expression in chronically-infected CD4+ T cells

**DOI:** 10.1186/1742-4690-11-S1-P114

**Published:** 2014-01-07

**Authors:** Hsin-Ching Lin, Peter Simon, Arnold B Rabson

**Affiliations:** 1 The Child Health Institute of New Jersey, USA; 2 The Cancer Institute of New Jersey, USA; 3 Department of Pharmacology, UMDNJ-RWJMS, New Brunswick, NJ, USA; 4 Department of Pathology and Laboratory Medicine, UMDNJ-RWJMS, New Brunswick, NJ, USA; 5 Department of Pediatrics, UMDNJ-RWJMS, New Brunswick, NJ, USA

## 

The pathogenesis of HTLV-1-associated diseases involves complex interactions between HTLV-1 and the infected host, and is dependent on viral replication and gene expression. Previous studies from our laboratory have shown that T cell receptor- and phorbol ester (PMA)-mediated stimulation of chronically infected CD4 T-cells increases the expression of integrated HTLV-1 proviruses. Detailed analysis of HTLV-1 RNA and protein species following PMA treatment of the FS and SP HTLV-1-infected T cells demonstrated rapid induction of tax/rex mRNA peaking prior to other HTLV-1 RNA species. This rapid increase in tax/rex mRNA was associated with markedly enhanced tax/rex mRNA stability. (from 3.5 hr to >24hr in treated cells), while the stability of unspliced or singly spliced HTLV1 RNAs did not increase. PMA treatment also increased RNA transcription. These data contrast with a delayed increase in HTLV-1 RNA transcription following treatment the HDAC inhibitor, SAHA. To further identify the elements and mechanisms responsible for enhanced tax/rex RNA stability, we adapted a luciferase reporter assay in which tax/rex cDNA sequences were linked to the 3’ end of luciferase gene. Increased luciferase activity was observed with this reporter plasmid upon PMA treatment, as compared with a reporter plasmid containing tax antisense sequences. Our data support a model whereby T cell activation leads to increased HTLV-1 gene expression through increased tax/rex mRNA stability, which in turn results in induction of Tax protein expression, and further activation of HTLV-1 gene expression, and associated changes in cellular gene expression, contributing to increased T cell survival and proliferation and disease.

